# The effect of intranasal oxytocin on neural response to facial emotions in healthy adults as measured by functional MRI: A systematic review

**DOI:** 10.1016/j.pscychresns.2017.11.017

**Published:** 2018-02-28

**Authors:** John Tully, Anthony S. Gabay, Danielle Brown, Declan G.M. Murphy, Nigel Blackwood

**Affiliations:** a Department of Forensic and Neurodevelopmental Sciences, Institute of Psychiatry, Psychology and Neuroscience, Kings College London, London, United Kingdom; b Department of Neuroimaging, Kings College London, London, United Kingdom; c Institute of Psychiatry, Psychology and Neuroscience, Kings College London, London, United Kingdom

**Keywords:** Empathy, Neuropeptides, Neurochemistry, Amygdala, Anterior cingulate cortex, Fusiform cortex, Biomarkers

## Abstract

Abnormalities in responses to human facial emotions are associated with a range of psychiatric disorders. Addressing these abnormalities may therefore have significant clinical applications. Previous meta-analyses have demonstrated effects of the neuropeptide oxytocin on behavioural response to facial emotions, and effects on brain, as measured by functional MRI. Evidence suggests that these effects may be mediated by sex and the role of eye gaze. However, the specific effect of oxytocin on brain response to facial emotions in healthy adults has not been systematically analysed. To address this question, this further systematic review was conducted. Twenty-two studies met our inclusion criteria. In men, oxytocin consistently attenuated brain activity in response to negative emotional faces, particularly fear, compared with placebo, while in women, oxytocin enhanced activity. Brain regions consistently involved included the amygdala, fusiform gyrus and anterior cingulate cortex. In some studies, oxytocin increased fixation changes towards the eyes with enhanced amygdala and/or fusiform gyrus activation. By enhancing understanding of emotion processing in healthy subjects, these pharmacoimaging studies provide a theoretical basis for studying deficits in clinical populations. However, progress to date has been limited by low statistical power, methodological heterogeneity, and a lack of multimodal studies.

## Highlights

• This review examines the effect of oxytocin on brain response to facial emotions in healthy adults.
• In men, oxytocin consistently attenuates brain activity in response to negative emotional faces, particularly fear.
• In women, oxytocin appears to enhance brain activity.
• Brain regions involved include the amygdala, fusiform gyrus and anterior cingulate cortex.
• In some studies, oxytocin increases eye gaze with enhanced amygdala +/- fusiform gyrus activation.

## 1 Introduction

Emotion recognition is a central component of social cognition. It provides the capacity to understand the intentions, feelings, and reactions of others (Shahrestani et al., 2013). Emotion recognition has been linked to altruistic helping behaviour (Marsh et al., 2007) and higher relationship quality with a lower rate of depression (Carton et al., 1999). In contrast, deficits in emotion recognition are associated with increased social anxiety and avoidance and antisocial behaviour as well as psychiatric disorders including schizophrenia, depression, autism spectrum disorders (ASD) and psychopathy (Chung et al., 2013; McClure and Nowicki Jr, 2001; McClure et al., 2014; Uljarevic and Hamilton, 2013). Furthermore, social cognitive deficits, including emotion recognition, predict social function in both early psychosis (Bertrand et al., 2007) and autism (Losh et al., 2009) above other tests of neurocognition. Emotion recognition has been investigated in functional neuroimaging studies by analysing neural response to facial emotions, alongside behavioural measures. Brain regions identified by meta-analyses of functional neuroimaging studies as having a role in response to facial emotions include globus pallidus, lateral orbitofrontal cortex, medial prefrontal cortex, subcallosal cingulate and most consistently, the amygdala, anterior cingulate cortex (ACC) and the insula. (Murphy et al., 2003, Phan et al., 2002).

Oxytocin is a neuropeptide central to the regulation of complex social cognition and behaviours, such as attachment, social exploration, and recognition. For example, in prairie voles, oxytocin plays a key role in pair bond formation (Young and Wang, 2004) and reducing fear in social interactions (Carter et al., 2008). In healthy humans, oxytocin binds to receptors in social brain regions such as the amygdala and ACC (Boccia et al., 2013) and plays a key role in social brain functions such as modulation of social stress, emotion recognition and memory formation (Meyer-Lindenberg et al., 2011). Oxytocin has also been shown to exert modulatory effects on brain activity in people with mental disorders. For instance, in women with borderline personality disorder, oxytocin attenuates abnormal behavioural and amygdala processing of emotional stimuli (Bertsch et al., 2013). In autism, some (Anagnostou et al., 2012, Andari et al., 2010, Guastella et al., 2010, Hollander et al., 2007), albeit not all (Dadds et al., 2014), studies have reported that oxytocin improves emotion recognition, and modulates activity in the medial prefrontal cortex (Aoki et al., 2015, Watanabe et al., 2013).

The impact of oxytocin on behavioural and brain response to facial emotions has typically been evaluated experimentally using a single dose of intranasal oxytocin given 30–45 min before behavioural experiments (Guastella and MacLeod, 2012). Some studies have suggested that oxytocin specifically improves the perception of happy faces, (Marsh et al., 2007, Schulze et al., 2011), while others report that oxytocin improves the recognition of angry, sad, and fearful emotions in faces (Ellenbogen et al., 2012, Fischer-Shofty et al., 2010; Lischke et al., 2012a). Guastella and MacLeod (2012) have argued that differences in methodologies or samples between studies may contribute to such inconsistent findings. It should also be noted that recent reviews of the oxytocin literature (Quintana, 2018; Walum et al., 2016) have highlighted that many studies of behavioural effects are underpowered, and have made recommendations to improve the reliability of oxytocin research and identify more promising avenues for future work. These include employing equivalence tests in statistical analysis (Quintana, 2018), performing a priori power calculations, disclosing methods and findings transparently, and working collaboratively to increase power and replicate findings (Walum et al., 2016).

Notwithstanding these concerns, a meta-analysis of the effects of single dose oxytocin on recognition of facial emotions in healthy subjects showed that intranasal oxytocin administration enhances emotion recognition of faces overall (Hedges g effect size 0.29) (Shahrestani et al., 2013). When analysis was restricted to facial expression types, significant effects of oxytocin on recognition accuracy were specifically found for the recognition of happy and fearful faces. A further recent meta-analysis demonstrated that a single dose of intranasal oxytocin significantly improved the recognition of basic emotions, particularly fear, among healthy individuals (Leppanen et al., 2017).

While these are interesting findings, neurocognitive or behavioural studies do not demonstrate underlying activity in the brain, the substrate for any potential therapeutic modulation. In contrast, using functional imaging to elucidate which brain regions are affected by oxytocin can demonstrate a neural underpinning for its effects. Applied to mental disorders, this approach may be used to demonstrate differential modulation of brain compared to healthy controls and help identify treatment targets and pathways into and pathways out of these disorders. Further, many neuroimaging studies utilise paradigms which measure ‘implicit’ processing of facial emotions. Such tasks require participants simply to identify the sex of faces presented, or stay focused on the face, rather than explicity identifying the emotion. This may have the added benefit of removing variance caused by participant effort on behavioural tasks (Bethlehem et al., 2013).

Furthermore, several studies have called into question the supposed ‘prosocial’ effects of oxytocin (Bartz et al., 2011, De Dreu et al., 2011a, De Dreu et al., 2010, De Dreu et al., 2011b) and suggested likely mediation of its effects by social context (Bartz et al., 2011) and other social (Heinrichs et al., 2003, Meinlschmidt and Heim, 2007, Saphire-Bernstein et al., 2011, Strathearn et al., 2009) physiological (Cardoso et al., 2013, Ditzen et al., 2009, Norman et al., 2012), and genetic (Feldman et al., 2013, Kim et al., 2010, Marsh et al., 2012, Rodrigues et al., 2009, Saphire-Bernstein et al., 2011, Tost et al., 2010, Tost et al., 2011, Walum et al., 2012) factors. Functional imaging may therefore also have a role in elucidating differential neural mechanisms underlying these confounding variables. Particularly relevant to processing of facial emotion recognition may be eye-gaze, which is an important factor in classifying facial expressions (Haxby et al., 2002, Smith et al., 2005) and has been shown to be affected by oxytocin (Domes et al., 2013) and mediated by the amygdala (Gamer and Büchel, 2009).

Two recent meta-analyses of FMRI studies have demonstrated that, in healthy controls, intranasal administration of oxytocin resulted in consistent alterations in activation of brain regions, including the amygdala, temporal lobes, ACC and insula, during the processing of a range of social stimuli, not exclusive to facial emotions (Wang et al., 2017, Wigton et al., 2015). The aim of our review was to systematically analyse the evidence specifically for modulation of neural processing of facial emotions by oxytocin, as measured by fMRI, in healthy controls. We discuss the specific effects of oxytocin on processing of individual emotions, as examined by some of the studies. We discuss mediation of oxytocin's effects by sex and specific emotion. We consider the potential impact of use of implicit vs explicit paradigms. We also consider apparent discrepancies in hemispheric lateralisation and brain regions identified across studies. Finally, we discuss the effects of oxytocin on gaze processing and the role of genetic polymorphisms, as examined by some of these studies.

## 2 Methods

### 2.1 Search strategy

We used a systematic search strategy following the PRISMA guidelines for systematic reviews (Moher et al., 2009) to identify relevant studies for inclusion in our review. The databases used were *MEDLINE (PubMed), Web of Science* and *The Cochrane Central Register of Controlled Trials* (CENTRAL) *Embase*, ClinicalTrials.gov, PsychInfo, BioMed Central, Google Scholar, and Scopus. Non-automated searching and cross referencing of relevant publications was also conducted. Search terms used were “Oxytocin” AND “fMRI OR functional magnetic resonance imaging” AND “facial emotion recognition OR empathy task OR emotion recognition OR facial expression OR valence task”. All studies identified as meeting the pre-specified eligibility criteria were exported to EndNote. Study abstracts were assessed for inclusion or exclusion by three authors (JT, AG, DB). Full papers were obtained for studies which met all inclusion criteria as outlined above. Papers published online up until September 2017 were included. Although no limits were placed on the language for an article, all articles found that fit our selection criteria were published in English.

### 2.2 Eligibility criteria

The primary outcome of interest for this review was the effect of oxytocin on response to facial emotions as measured by functional magnetic resonance imaging (fMRI) in normal adult subjects. Studies were selected on the basis that (i) participants were administered a single dose of oxytocin intranasally, (ii) the effect of intranasally administered oxytocin on facial emotion processing was measured using fMRI, and (iii) a facial emotion processing paradigm (or paradigms) were used to measure the effects of oxytocin on response to facial emotions. We included studies using paradigms measuring both implicit and explicit responses to facial emotions. Studies considered for selection were randomised controlled trials (RCT), cross-over studies, between-subject design studies and within-subjects design studies. Only normal population adult subjects were included. Studies that used a patient population but also reported findings for healthy controls were included if they satisfied all other criteria. There were no restrictions on sex. Exclusion criteria were postmortem studies, structural imaging techniques, studies looking at only endogenous oxytocin or modes of delivery other than intranasal, and studies examining response to facial emotion tasks only (i.e. without fMRI).

### 2.3 GRADE scoring

To evaluate the quality of the evidence, GRADE scoring (Guyatt et al., 2008) was employed. The GRADE system evaluates quality across studies and specifically assesses methodological flaws within the component studies, consistency of results across different studies, generalisability of research results to the wider patient base and how effective the treatments have been shown to be.

## 3 Results

### 3.1 Samples, demographics, and study design

**Fig. 1:**
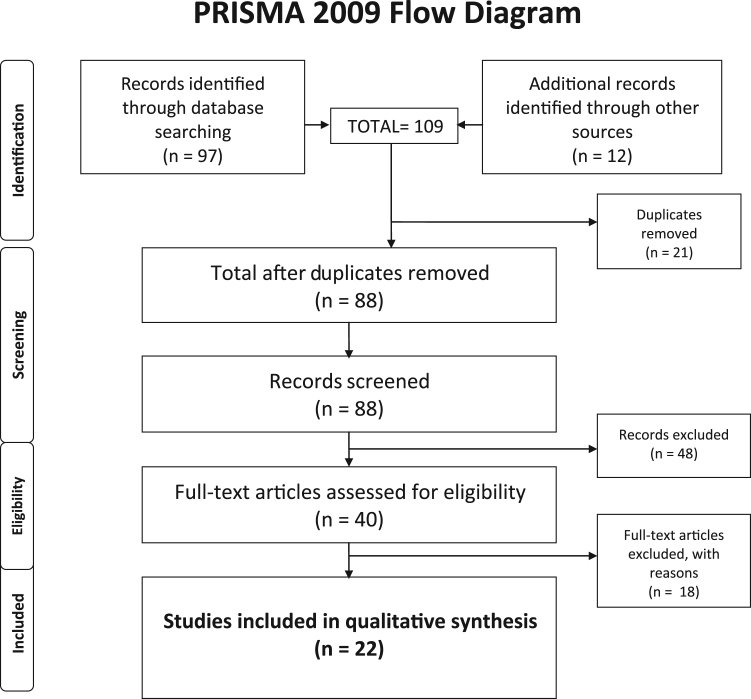
Study selection process (PRISMA).

The results from the literature search are illustrated in the PRISMA diagram (Fig. 1). Healthy control samples were male (n = 16 studies), female (n = 4), or both male and female (n= 2) participants without histories or signs of neurological, endocrine, psychiatric or serious somatic illnesses and not taking psychotropic medications.

Samples, demographics and key findings are outlined in Table 1. Sample ages ranged on average from 19.66 years (± 1.45) to 39.3 years (± 6.2). Sample sizes ranged from 13 to 116 participants with a total of 772 subjects from the combined studies (178 females) and an average group size of 36.54 (note, two studies by Koch et al., 2016a, Koch et al., 2016b used the same sample, as did the studies by Sauer et al., 2012 and Montag et al., 2013). Sixteen studies examined healthy populations only, while seven studied clinical populations but included healthy controls. Of the twenty-two studies included, ten used a double-blind, placebo controlled within-subject design and eleven used a double-blind, placebo-controlled between-subject design. Spengler et al. (2017) used a between-subjects design to compare doses and a crossover design to compare times of dosing. Thirteen studies used a paradigm measuring explicit response to facial emotions, while eight used a paradigm measuring implicit response; Voorthuis et al. (2014) compared explicit to implicit responses. Only seven studies used neutral facial expressions as a specific contrast to emotional faces. The most frequently studied emotions were happiness (seventeen studies), fear (fourteen) and anger (eleven). Sadness, surprise, and disgust were each studied in two studies only.

**Table 1:** Details of included studies.

**Study**	**Subjects (mean age and s.d.)**	**Study design**	**Intranasal Oxytocin application**	**Facial emotion response paradigm and design**	**Facial Emotions presented in task**	**Summary of FMRI facial emotion paradigm and*****functional connectivity findings***
Bertsch et al. (2013)	41 females (24.4/24.6 +/- 3.9/4/4 years (oxytocin/placebo))	Double-blind, placebo controlled ***between- subject*** design	26 IU; 45 mins before fMRI	**Explicit** classification of 72 briefly presented faces, unambiguously depicting expressions	Happiness, fear, anger	OT enhanced right, centrally located amygdala activation in response to fearful faces
						
Domes et al. (2007)	13 Males (25.7 ± 2.9 years)	Double-blind, placebo controlled **within-subject** design	24 IU; 45 mins before task	**Implicit** Facial Affect Recognition Paradigm	Happiness, fear, anger	ROI: OT reduced activity of the right amygdala in response to happy, fearful and angry faces
						WBA: modulatory effects of OT in prefrontal and temporal areas as well as in the brainstem
				Block design		
						
Domes et al. (2010)~	16 Females (24.2 ± 2.5 years)	Double-blind, placebo controlled **within-subject** design	24 IU; 45-60 mins before task	**Explicit** rating of labelled emotions	Happiness, fear, anger (and neutral)	OT enhanced activity in the left amygdala, FG and STG in response to fearful faces and in inferior frontal gyrus in response to angry and happy faces
				Block design		
						
Domes et al. (2014)	14 Males (23.6 ± 5.4 years)	Randomized double-blind, placebo-controlled, **within-subject**, cross-over design	24 IU; 45 mins before start of fMRI experiment	**Explicit** rating of facial emotion based on eyes or mouth	Happiness, fear, anger, disgust, sadness, surprise	No observed effects in amygdala ROI analysis; OT administration Increased activation in inferior frontal gyrus (for eye stimuli) and fusiform gyrus (for mouth stimuli) on WBA
				Event-related design		
						
Gamer et al. (2010)~	46 Males (25.0 ± 3.7 years)	Double-blind, placebo controlled ***between-subjec***t design	24 IU; 45 mins before task	**Explicit** classification of emotions	Happiness, fear (and neutral)	OT attenuated activation in lateral and dorsal regions of the anterior amygdala for fearful faces but enhanced activity for happy expressions
				Event-related design		
						
Gorka et al. (2015)	17 Males (29.9 years)	Randomized double-blind, placebo-controlled, **within-subject design**	24 IU; 45 mins before start of fMRI experiment	EFMT: **explicit** matching of emotion shown with one of two others shown	Happiness, fear, anger	*OT reduced amygdala connectivity to insula and mid/dACC (in healthy controls)*
				Block design		
						
Groppe et al. (2013)	28 females (26.64 ± 5.55 years)	Randomized, double-blind, placebo-controlled ***between subject (parallel group***) design	26 IU; 30 min before task	Social incentive delay task- uses happy or angry faces as social cues for reward; recognition of emotions **implicit**	Happiness, anger	OT led to significantly stronger activation in VTA to cues of high social reward (happy faces) but not low social reward (angry faces) compared with control cues
						
Kanat et al. (2015a)	50 males (placebo 23.9 ± 2.74, OT 24.32 ± 3.43)	Randomised, double-blind, placebo-controlled, ***between-subjects*** design	24 IU; 55 min before task	**Implicit** response to fearful or happy faces, and fearful or happy masked eyes	Happiness, fear	ROI: no effect during the processing of fearful as compared with happy control stimuli; oxytocin significantly reduced right amygdala responses to fearful as compared with happy stimuli in the eye condition
				Block design		
						WBA: oxytocin reduced brain responses to fearful as compared with happy stimuli in face and eye stimuli within the left ACC and left MTG
						
Kanat et al. (2015b)	49 males (23.64 years ± 2.81)	Randomised, double-blind, placebo-controlled, ***between-subjects*** design	24 IU; 45 mins before task	**Explicit** identification of emotion	Happiness, fear	ROI: OT potently reduced left and right amygdala responses to eye region of masked angry faces & dampened amygdala reactivity to mouth as compared to the eye region of masked happy faces.
				Block design		
						WBA: OT specifically reduced the response of the fusiform gyrus to anger cues from the eye region; OT attenuated reactivity to masked threat from the eyes as compared to the mouth within brain stem regions and the striate cortex
						
Kirsch et al. (2005)	15 Males (26.7 ± 3.0 years)	Double-blind, placebo controlled **within-subject** design	27 IU; 50 mins before task	Facial emotion recognition task (Hariri)- **explicit** matching of emotions	Fear, anger	*OT decreased amygdala connectivity to the brain stem*, & decreased left amygdala activation in response to angry & afraid faces.
				Block design		
						
Koch et al., 2016a, Koch et al., 2016b* **a= Task b= Functional Connectivity**	20 Males (41.35 ± 10.62years); 20 Females (38.65 ± 9.48 years)	Randomized, double-blind, placebo-controlled, cross-over study **(within-subject** design**)**	40 IU; 45 mins before task	Facial emotion recognition task (Hariri)- **explicit** matching of emotions	Happiness, fear, anger	No significant main effect of OT on left and right amygdala reactivity towards emotional faces
						*Decreased right BLA to left dACC connectivity in males*
				Block design		
						
Labuschagne et al. (2012)	18 Males (29.9 ± 10.2 years)	Randomized, double-blind, placebo-controlled **within-subjects** design	24IU; 50 mins before FMRI	**Explicit** rating of positive, negative or neutral	Happiness, sadness (and neutral)	Sad vs. neutral faces: OT decreased activity in the right ACC, right MFG, right premotor and left SPC, and enhanced activity in the thalamus.
				Block design		
						Happy vs neutral: OT suppressed activity in right mPFC, right ACC, left MFG, left precuneus, left FG, left CF and cerebellum; enhanced right STG
						
Luo et al. (2017)	43 males, 43 females (22.41 ± 2.1 years)	Randomized, double-blind, placebo-controlled ***between-subject*** design	24IU; 45 mins before task	**Implicit-** rapid response gender-discrimination task	Happiness, fear, anger, sadness, disgust	OT suppressed IFG, dorsal ACC and anterior insula responses to threatening face stimuli in men, but increased them in women.
				Event-related design		
						*Sex-dependent effects on amygdala, ACC and IFG functional connectivity that were mainly driven by reduced coupling in women following OXT*
						
^+^Montag et al. (2013)~	*M* = 24.9 ± 2.6 years	Double-blind, placebo controlled ***between-subjects*** design	25 IU; 45 mins before task	**Implicit-** gaze direction identification	Neutral (direct vs indirect gaze)	Genetic variation of the OXTR gene (rs401015) modulated right amygdala activity for direct > averted gaze under influence of OT
						
Petrovic et al. (2008)~	27 Males**	Double-blind, placebo controlled ***between-subject*** design	32 IU; 45 mins before task	**Implicit-** aversive conditioning procedure	Neutral (direct vs indirect gaze)	Effect of OT associated with an attenuation of activity in anterior medial temporal and anterior cingulate cortices.
				Random Block Design/Mixed Design		
						
Quintana et al. (2016)	18 Males (23.81 ± 3.33 years)	Double-blind, placebo controlled crossover (**within subjects** design)	8IU vs 24 IU; 40 mins before task	**Explicit** ratings of degree of emotion in faces	Happiness, anger (vs neutral)	8 IU dose: OT led to significantly reduced right amygdala activation in response to angry and happy faces 24 IU dose: no significant findings vs placebo
				Block design		
						
Radke et al. (2017)	57 Males (22.4 ± 3 years)	Randomized, placebo-controlled, double-blind ***between subjects*** design	24 IU; 45 mins before task	**Explicit** recognition of faces	Happiness, anger	OT decreased right amygdala activation during approach (but not avoidance) of angry faces
				Block design		
						*No OT induced alterations in connectivity between the right aPFC and the amygdala*
						
^+^Sauer et al. (2012)~	56 Males***	Double-blind, placebo controlled ***between-subjects*** design	25 IU; 30 mins before the	**Explicit**: extended version of Facial emotion recognition task (Hariri)	Happiness, fear, anger	CC genotype showed higher activation of left fusiform gyrus during visual processing of social stimuli.
			start of fMRI experiment			
						Under OT activation differences between genotypes were more evident
				Block design		
						
Shin et al. (2015)	16 Males (31.3 ± 7.6)	Randomized, double-blind, placebo-controlled crossover **(within subjects)** design	40 IU; 45 mins before FMRI scan	**Implicit** responses to emotional faces	Happiness, fear (vs neutral)	Overall, OT increased bilateral amygdala activation in controls, however posthoc analysis showed attenuated activation in response to the fearful faces and increased activation in response to happy faces
				Block design		
						
Spengler et al. (2017)	116 Males (24.7 ± 4.4 years)	Randomized, double-blind, placebo-controlled ***between subjects*** (dose) crossover (time) design	Varied- 12/24/48 IU and 15/45/75 mins before task	**Explicit** responses to morphed emotional faces	Happiness, fear (vs neutral)	24 IU Dose: significantly reduced the response to fearful faces in the left amygdala; high intensity emotion response only significant at 45 minutes
				Event-related design		
						
Voorthuis et al. (2014)	50 Females (19.66 ± 1.45 years)	Randomised, placebo-controlled, ***between subjects** design*	16 IU; 50-60 mins before task	iFEEL paradigm- tested **implicit vs explicit** responding to infant emotional faces	Not specified	OT increased activation in IFG, MTG and STG in emotion recognition vs gender recognition (but decreased performance on the IFEEL picture task)
						
Wittfoth-Schardt et al. (2012)	19 Males (39.3 ± 6.2 years)	Randomized, double-blind, placebo-controlled **within-subjects** design	24 IU; 30 mins before FMRI	**Implicit** responses- asked to attentively view only	Friendly- level of attachment varied (own child vs other child)	*OT reduced activation and functional connectivity of the left GP with reward- and attachment-related regions responsive to pictures of children’s faces*
				Event-related design		

**Key**

aPFC= anterior prefrontal cortex; BOLD= Blood oxygenation level-dependent; CF= calcarine fissure; EFMT= Emotional Face Matching Task; EPI= Echo planar imaging; FG= fusiform gyrus; fMRI= Functional magnetic resonance imaging; FEW= Family-wise error; FWHM= Full-width at falf-maximum; GLM= General linear model; GP= Globus pallidus; IFG= Inferior frontal gyrus; OT= Oxytocin; PL= Placebo; MDBF = multidimensional mood questionnaire; MFG= Middle frontal gyrus; MTG= midtemporal gyrus; mPFC= medial prefrontal cortex; PSC= Percent signal change; REM= Random-effects model; ROI= Region of Interest; SPC= superior parietal cortex ; SPM= Statistical parametric mapping; STG= superior temporal gyrus; T= Tesla; VTA= ventral tegmental area; WBA= Whole Brain Analysis.

* All ‘trauma-exposed’ controls; **OT group *n* = 15 (mean age 25.5 years) Placebo group *n* = 12 (mean age 24.2 years); ***CC genotype: n=30, mean (25.2±2.76 years); CA/AA genotype: n=25 (24.6±2.42 years); ^+^ = These two studies had the same sample, both were imaging-genetics studies; ~ = examined effect of eye-gaze/tracking.

### 3.2 Key findings in relation to brain region, emotion type, sex and implicit vs explicit paradigms

In the first study of its kind, Kirsch et al. (2005) demonstrated that oxytocin decreased amygdala activation in response to angry and fearful faces in men. Several studies since have supported the hypothesis that oxytocin attenuates brain activity in response to negative facial emotions (fear, anger, disgust and sadness) in male samples. For example, oxytocin reduced activity of the right amygdala in response to fearful and angry faces (Domes et al., 2007); attenuated activation in lateral and dorsal regions of the anterior amygdala (Gamer et al., 2010) right amygdala, left ACC, left midtemporal gyrus (MTG) (Kanat et al., 2015a) and left amygdala (Spengler et al., 2017) in response to fearful faces/stimuli (eyes); reduced left and right amygdala responses to angry stimuli (Kanat et al., 2015b); and decreased activity in the right ACC, right middle frontal gyrus, right premotor and left superior parietal cortex in response to sad vs neutral faces (Labuschagne et al., 2012). Luo et al. (2017) showed that oxytocin reduced activity in left and right ACC, left and right inferior frontal gyrus (IFG) to fearful faces, and left and right IFG to angry faces and sad faces, all with a neutral contrast.

In contrast, in three studies with female subjects, oxytocin *increased* brain activity in response to negative emotions. Domes et al. (2007) demonstrated that oxytocin enhanced activity in the left amygdala, fusiform gyrus and superior temporal gyrus in response to fearful faces and in inferior frontal gyrus in response to angry faces in healthy women, while Bertsch et al. (2013) demonstrated enhanced right, centrally located amygdala activation in response to fearful faces with oxytocin in their healthy controls. Luo et al. (2017) demonstrated increased activity in response to oxytocin in right IFG to angry faces, left ACC, left insula and left and right IFG to fearful faces, and left and right IFG to sad faces. A notable exception to the patterns in males and females was the study by Koch et al. (2016b), which showed no significant main effect of oxytocin on left and right amygdala reactivity towards emotional faces in either sex.

Findings for brain responses to positive emotional faces (happiness) were more equivocal. In studies in men, oxytocin variously reduced activity of the right amygdala (Domes et al., 2007; Quintana et al., 2016), bilateral amygdala (Shin et al., 2015), and right medial prefrontal cortex, right ACC, left middle frontal gyrus, left precuneus, left fusiform gyrus, left calcarine fissure and cerebellum (Labuschagne et al., 2012) in response to happy faces, but *enhanced* activation in lateral and dorsal regions of the anterior amygdala (Gamer et al., 2010) and right superior temporal gyrus (Labuschagne et al., 2012). In females, oxytocin led to significantly stronger activation of the ventral tegmental area to cues of high social reward (happy faces) and enhanced IFG in response to happy faces (Domes et al., 2010). Outcomes based on implicit vs explicit facial emotion paradigms are shown in Table 2, and implications are discussed below.

**Table 2:** Findings in relation to implicit vs explicit paradigms.

*Modulation of:*	**Effect of oxytocin with Implicit paradigm Males**	**Effect of oxytocin with Implicit paradigm Females**	**Effect of oxytocin with Explicit Paradigm Males**	**Effect of oxytocin with Explicit Paradigm Females**
*Amygdala*	**Yes**		**Yes**	**Yes**
	Domes et al. (2007) (↓, right)		Gamer et al. (2010) (↓, right to fear, ↑ to happiness)	
	Kanat (2015a) (↓, right)		Kanat et al. (2015b) ROI: OT potently ↓ left and right)	
	Shin (↓, right to fear, ↑ to happiness)		Kirsch et al. (2005) (↓ left)	Bertsch et al. (2013) (↑, right)
	Radke (↓, right)		Quintana et al. (2016) (↓, right)	Domes et al. (2010) (↑, left)
	Montag et al. (2013) (see Table 1)		Radke et al. (2017) (↓, right)	
			Spengler et al. (2017) (↓, left)	
	**No**	**No**	**No**	**No**
	Domes et al. (2014)	Groppe et al. (2013)		
	Petrovic et al. (2008) (aversive conditioning procedure)		Koch et al. (2016a) (↓, right)	Koch et al. (2016a)
	Montag et al. (2013) (gaze task)			
	Sauer et al. (2012)			
*Cortical/other*	Domes et al. (2007) (prefrontal, temporal, brainstem)			Domes et al. (2010) (FG and STG, IFG)
	Domes et al. (2014) (IFG, FG)	Groppe et al. (2013) (VTA)	Kanat et al. (2015b) (FG, brainstem, striatecortex)	
	Kanat et al. (2015a) (ACC, MTG; fear)	Luo et al. 2017 (IFG, dACC, anterior insula)	Labuschagne et al. (2012) (multiple, see Table 1)	
	Koch et al. (2016a)			
	Luo 2017 (IFG, dACC, anterior insula)			
	Petrovic et al. (2008) (temporal and ACC)			
*Functional connectivity*	Wittfoth-Schardt et al. (2012)		Gorka et al. (2015)	Luo et al. 2017.
	(↓ left GP with reward regions)		(↓ amygdala to insula and mid/Dacc0 Kirsch et al. (2005) (↓amygdala connectivity to brainstem0	(↓ amygdala, ACC and IFG)
			Koch et al. (2016) (↓ right BLA to left dACC connectivity)	

### 3.3 Functional connectivity and eye gaze

Kirsch et al. (2005) first demonstrated that oxytocin decreased amygdala connectivity to the brain stem in response to fearful and angry faces in healthy subjects. Other studies including healthy subjects have since demonstrated reduced connectivity in response to oxytocin when responding to emotional faces. When processing happy, fearful and angry faces, Gorka et al. (2015) demonstrated that oxytocin reduced amygdala connectivity to insula and mid/dorsal ACC, while Koch et al. (2016a) demonstrated that oxytocin decreased right basolateral to left dorsal ACC connectivity in males. Wittfoth-Schardt et al. (2012) showed that oxytocin reduced functional connectivity of the left globus pallidus with reward- and attachment-related regions responsive to pictures of children's faces. Luo et al. (2017) showed sex-dependent effects on amygdala, ACC and IFG functional connectivity that were mainly driven by reduced coupling in women following oxytocin. However, Radke et al. (2017) found no oxytocin-induced alterations in connectivity between regions examined (right anterior prefrontal cortex and amygdala).

Five of the studies identified for this review examined the effect of eye gaze on outcome measures. In males, Petrovic et al. (2008) demonstrated abolition by oxytocin of differential negative affective ratings in faces exposed to an aversive conditioning. In amygdala and fusiform gyrus, this modulation was stronger for faces with direct gaze, relative to averted gaze, consistent with a relative specificity for socially relevant cues. Gamer et al. (2010) demonstrated that oxytocin increased the likelihood of reflexive gaze shifts toward the eye region irrespective of the depicted emotional expression. Sauer et al. (2012) showed that genotype (of OXTR) led to differential activation of left fusiform gyrus activation during a gaze processing task. Analysing both substance conditions separately led to a higher activation for A- carriers compared with A+ under oxytocin, but not under placebo. Using the same sample, in a further study exclusively examining the effect of oxytocin on eye gaze, Montag et al. (2013) showed that a separate genetic variation of the OXTR gene (rs401015) modulated right amygdala activity for direct vs averted gaze under influence of oxytocin. This interaction was due to increased activation to directed gaze and decreased activation to averted gaze under oxytocin in TC carriers. In females, oxytocin resulted in enhanced right, more centrally located amygdala activation in response to fearful faces with initial fixation on the eye region in those in the oxytocin condition. Only Domes et al. (2010, also in females) failed to demonstrate a differential effect of eye gaze; their observed effects of oxytocin were independent of fixation pattern to specific sections of the facial stimuli as revealed by eye tracking.

### 3.4 GRADE scoring

An overall GRADE score of 1 (very low) was allocated to the current review. This was based on:

– Type of evidence: evidence provided by RCTs (+4);
– Quality: problems with two ‘quality’ elements (sparse data and methodological concerns) (−2);
– Consistency: problems with one ‘consistency’ element (lack of agreement between studies) (−1).
– Directness: population and outcomes broadly generalizable (0);
– Effect size: not all effect sizes >2 or <0.5 and significant; or if OR/RR/HR not significant (0).

## 4 Discussion

The aim of this review was to examine the evidence for modulation by oxytocin of neural processing of responses to facial emotions, as measured by fMRI. Previous reviews on the effects of oxytocin on response to facial emotion have focussed on effects on task performance only (Leppanen et al., 2017, Shahrestani et al., 2013), or more broadly on oxytocin-neuroimaging studies (Bethlehem et al., 2013, Kanat et al., 2014). Meta-analyses examining the effect of oxytocin on specific brain regions (Wang et al., 2017, Wigton et al., 2015) have discussed response to facial emotions but have not separately analysed neuroimaging data for the effect of oxytocin specific to facial emotion response tasks. Given the importance of neural processing of facial emotion to emotion recognition and social cognition, we believe the specific effects of oxytocin in healthy subjects was an important consideration warranting further exploration and therefore performed this review. Further, we wished to consider differential outcomes with the use of implicit vs explicit paradigms for facial emotion recognition. Finally, we wished to consider mediating roles of eye gaze, which was examined in some of the studies, and has been suggested to play an important role in processing of facial emotion, and polymorphisms of OXTR, which are thought to influence the effect of oxytocin.

As expected, there were significant effects of oxytocin in all studies identified, with evidence for modulation by oxytocin of key social brain regions, including during processing of direct vs indirect eye gaze. Also as expected, given previous research on oxytocin, these effects were mediated by other factors, including sex and emotion type, in many of the studies. Comparison of implicit vs explicit paradigms however did not reveal a clear pattern of differential effects.

### 4.1 Differential effects of oxytocin relative to brain region and hemisphere

The most frequently identified region for effects of oxytocin on brain activity was the amygdala (twelve studies). This was not unexpected, given the key role the amygdala has been shown to play in social brain function (Adolphs, 2010, Heim et al., 2009) and evidence for direct action of oxytocin in the amygdala (Heinrichs et al., 2009, Landgraf and Neumann, 2004). It is worth noting however that all of these studies, except Domes et al. (2010), used the amygdala for region of interest (ROI) analysis, which may have biased the results. In contrast, other brain regions were mostly identified when whole brain analysis (WBA) was employed.

In three studies (Domes et al., 2010; Kirsch et al., 2005; Spengler et al., 2017), effects were observed exclusively in the left amygdala; in five (Bertsch et al., 2013; Domes et al., 2007; Kanat et al., 2015a; Quintana et al., 2016; Radke et al., 2017), effects were observed exclusively in the right amygdala; while in three others (Gamer et al., 2010; Kanat et al., 2015b; Shin et al., 2015) effects were observed in bilateral amygdalae. There is some evidence to suggest lateralisation of amygdala in relation to social brain function in humans. For example, (Murray, 2007) suggested a role for the left amygdala in the reward system. A review of previous studies of the role of the amygdala in facial emotion recognition, while yielding some apparently conflicting results, concluded that unconscious (masked) processing is mediated more readily by the right amygdala, and conscious processing more readily by the left amygdala (Markowitsch, 1999). This factor may help to explain the apparent discrepancy across studies, as all three studies with findings exclusively in left amygdala used explicit paradigms (though studies with significant findings in right and bilateral amygdalae used both implicit and explicit tasks). In relation to specific type of emotion, Lanteaume et al. (2007) found that the left amygdala, when stimulated, induced positive emotions, while the right amygdala induced negative emotion. This would not explain the disparity of findings in our review however, as activations were found for both positive and negative emotions in both left and right amygdalae.

Gamer et al. (2010) report more specific neuroanatomical localisation within the amygdala than in the other studies, identifying lateral and dorsal regions of the anterior amygdala as having localised activity. In healthy humans, functional neuroimaging studies have helped elucidate differential roles for amygdala subregions in different sensory modalities (Morris et al., 2001), different valences of auditory stimuli (Ball et al., 2007, Frühholz and Grandjean, 2013), functional connectivity in generalised anxiety disorder (Etkin et al., 2009), and response to SSRIs in social anxiety disorder (Faria et al., 2012). In psychopathy, a model of differential amygdala activation has been described in which the basolateral amygdala (BLA) is underactive while the activity of the central amygdala (CeA) is of average to above average levels (Moul et al., 2012). Related to facial emotion recognition, Goosen**s** et al., localised facial expression-evoked signal changes bilaterally in the superficial amygdala, in contrast to non-social visual stimuli (Goossens et al., 2009), suggesting that this subregion selectively extracts the social value of incoming sensory information. Hence, differentiating simply between right and left amygdala response may not be sufficiently discriminating in relation to effects of oxytocin on brain function. Further, oxytocin also appears to reduce connectivity between the amygdala and both cortical (insula and mid/dorsal ACC; Gorka et al., 2015) and brainstem (Kirsch et al., 2005) regions in healthy men, suggesting that oxytocin's effects may be in part through modulation of both top-down and bottom-up processes involving the amygdala.

The fusiform gyrus was also implicated in a number of studies (Domes et al., 2014; Domes et al., 2010; Kanat et al., 2015b; Petrovic et al., 2008; Sauer et al., 2012). It has been suggested that the role of the fusiform gyrus is specific to facial emotion recognition (Parvizi et al., 2012) and this has been the most consistently activated brain region in response to tasks incorporating facial stimuli (Wigton et al., 2015). Other regions of the temporal lobe were also implicated, including the anterior and medial temporal lobe (Petrovic et al., 2008), the superior temporal gyrus (Domes et al., 2010; Voorthuis et al., 2014) and the middle temporal gyrus (Domes et al., 2007; Domes et al., 2010; Voorthuis et al., 2014; Wittfoth-Schardt et al., 2012). As the meta-analysis by Wigton et al. (2015) demonstrated, overall, oxytocin appears to have sex-mediated effects in the temporal lobes, with increased activation in the temporal lobes in women and generally attenuated activation in the temporal lobes in men.

The anterior cingulate cortex (ACC) was impacted by oxytocin in two studies (Kanat et al., 2015a; Labuschagne et al., 2012). While the ACC plays an important role in cognitive tasks such as choice prediction (Kennerley et al., 2011), it has also been shown to be recruited in tasks such as emotional recall imagery and emotional tasks with cognitive demand (Phan et al., 2002). Oxytocin attenuated ACC activity in response to negative emotions in both studies, suggesting oxytocin influences top-down cortical modulation of response to emotional faces in the ACC. Activity in the insula has also been previously associated with emotion regulation (Phan et al., 2002), and left insular hyperactivation after oxytocin administration has been demonstrated by a recent meta-analysis (Wigton et al., 2015). However, the role of the insula appears to be primarily related to modulation of risk prediction and reward anticipation in a social context (Wigton et al., 2015). In our review, only one study (Domes et al., 2010) implicated the insula, demonstrating left insular activation in response to fearful, angry and happy faces. This study was with female subjects, but had a small sample (n = 16), and so inferences about sex-specific effects of oxytocin on the insula in processing facial emotion are of limited value.

Taken together, these studies emphasise the importance of a more nuanced understanding of brain regions involved in facial emotion recognition, particularly the amygdala. As functional imaging methodology and resolution continues to develop, improved anatomical specificity of functional imaging may help resolve uncertainties about specific effects of oxytocin on individual emotions. A further consideration is the use of ROI analysis in these studies. Given that many of the studies implicated regions other than the amygdala as being important to oxytocin's effects, it no longer seems appropriate to identify the amygdala as the sole region of interest in future study designs.

### 4.2 Differential effects of oxytocin relative to emotion, sex and implicit vs explicit paradigms

Oxytocin's effects on the brain appear to be dependent in part on the specific emotion in question. In all studies involving male subjects that reported neural responses to facial expression of specific emotions, except one (Domes et al., 2014), oxytocin reduced amygdala activity in response to fearful faces. This is in keeping with meta-analyses of functional neuroimaging studies in normal subjects, which have consistently demonstrated involvement of the amygdala in processing of fear (Murphy et al., 2003, Phan et al., 2002). One possible explanation for modulation by oxytocin is an anxiolytic effect, which has been demonstrated by numerous animal (Amico et al., 2004, Engelmann et al., 1999, Ring et al., 2006, Waldherr and Neumann, 2007, Yoshida et al., 2009) and some human (Guastella et al., 2009, Labuschagne et al., 2010) studies, and is likely to be amygdala-mediated (Viviani et al., 2011, Viviani et al., 2010). This would suggest a potential modulatory role for oxytocin in impaired regulation of facial emotion processing in anxiety disorders, which has been demonstrated in both adults (Demenescu et al., 2010, Goldin et al., 2009) and adolescents (McClure et al., 2014). Indeed, in their subjects with generalised anxiety disorder, Labuschagne et al. (2012) demonstrated that oxytocin significantly reduced heightened activation at baseline in the mPFC/ACC regions to levels similar to that of controls. Furthermore, as impaired fear recognition is thought to be an important factor in antisocial personality disorder (with and without psychopathy) (Blair, 2006, Dolan and Fullam, 2006, Marsh and Blair, 2008), modulation by oxytocin may also have therapeutic value for these disorders. This may be particularly relevant to individuals with antisocial personality disorder without psychopathy, who demonstrate a hyperactive amygdala at baseline (Blair, 2010).

The pattern of attenuation of amygdala responsivity by oxytocin in response to fearful faces extended to angry (Domes et al., 2007; Kanat et al., 2015a; Kirsch et al., 2005) and sad (Labuschagne et al., 2012) faces in other studies in males. In contrast, in response to happy faces, findings for the effects of oxytocin on the amygdala were mixed, with both attenuation (Domes et al., 2007; Quintana et al., 2016; Shin et al., 2015) and enhancement (Gamer et al., 2010; Labuschagne et al., 2012) being demonstrated. This apparent discrepancy reflects inconsistencies from behavioural studies (Shahrestani et al., 2013) and warrants further exploration. Differences in facial emotion paradigms may be a contributory factor, as discussed below.

Oxytocin's effects also appear to be mediated by sex differences. In females, as in males, enhancement of activity was seen in response to happy faces (Domes et al., 2010; Groppe et al., 2013). However, in contrast to males, oxytocin also enhanced neural activity in the amygdala (Bertsch et al., 2013; Domes et al., 2010) and superior temporal gyrus and fusiform gyrus (Domes et al., 2010) in response to fearful faces, and in inferior frontal gyrus in response to angry faces (Domes et al., 2010). Luo et al. (2017) showed that while oxytocin suppressed brain activity in inferior frontal gyrus, dorsal anterior cingulate and anterior insula in response to threatening face stimuli in males, it increased them in females. Further, this study showed further sex-dependent effects in functional connectivity (see Table 1) that were mainly driven by reduced coupling in women following oxytocin.

There are several potential explanations for the apparent differential effects of oxytocin on response to facial emotion in men and women. Although oxytocin modulates both salience and motivational aspects of social cues in both sexes (McCall and Singer, 2012), sex has been shown to moderate the effect of oxytocin on social judgments (Hoge et al., 2014), autonomic nervous system and emotional responses to couple conflict (Ditzen et al., 2012), and amygdala response to social judgements (Gao et al., 2016). Hence, sex is also likely to modulate response to facial emotions, another form of social cue. Further, meta-analysis has demonstrated differences in amygdala responsivity to emotions between men and women at baseline, with women demonstrating greater activation than men in response to negative emotions, particularly in the amygdala, and men exhibiting greater activation than women in the left amygdala, and other regions (Stevens and Hamann, 2012). Also, higher levels of progesterone in women (especially in luteal phase of menstrual cycle), are associated with enhanced sensitivity to fearful faces at baseline, and it may be that intranasal oxytocin further enhances vigilance to signals of potential threat (Domes et al., 2010). Finally, differences in the sensitivity of the oxytocin system to exogenous oxytocin between women and men could be due to modulations by gonadal steroids. Women differ from men in the luteal phase with regard to gonadal steroid hormones (Hawkins and Matzuk, 2008), thus providing a plausible explanation for inconsistent findings between men and women.

Debate persists about whether implicit or explicit responses to emotion are optimal measures of facial emotional processing, and many argue that they should be conceptualised and measured separately (Braunstein et al., 2017, Kliemann et al., 2013). Further, evidence suggests that implicit and explicit processing may have distinct neural substrates, with amygdala and subcortical limbic activity important for the implicit process and the response of the prefrontal cortex for the explicit process (Critchley et al., 2000, Xiao et al., 2016). Our analysis of outcomes based on implicit vs explicit facial emotion paradigms (Table 2) did not reveal any consistent pattern of findings. Most notably, amygdala activation was shown in both males and females using implicit tasks and explicit tasks, while no amygdala activation was observed in other studies using implicit (Domes et al., 2014; Groppe et al., 2013) or explicit (Koch et al., 2016b) tasks. Currently, evidence for differing patterns of oxytocin modulation based on type implicit vs explicit paradigm remains equivocal. Future studies may benefit however from delineating implicit and explicit brain response.

### 4.3 Differential effects of oxytocin relative to gaze processing

Eye gaze is a central component of identification of human facial emotion (Haxby et al., 2002, Smith et al., 2005). Impairments in eye gaze have been associated with deficits in facial emotion processing, particularly in autism (Guastella et al., 2008). While both averted- and direct-gaze faces are ‘social’ stimuli, in direct gaze, attention is toward the individual, while in averted gaze, the attention is directed toward an extrapersonal spatial location. Thus, it has been suggested that direct gaze is more socially relevant (Haxby et al., 2002; Petrovic et al., 2008). Intranasal oxytocin has been shown gaze specifically toward the eye region of human faces (Guastella et al., 2008), while a more recent study demonstrated that oxytocin differentially modulates eye gaze to social signals (Domes et al., 2013).

Four of the six studies which examined eye gaze processing studies showed an influence of oxytocin on related brain activity. Three (Bertsch et al., 2013; Gamer et al., 2010; Petrovic et al., 2008) showed modulation of the amygdala, while two (Petrovic et al., 2008; Sauer et al., 2012) implicated the fusiform gyrus. Together, these findings are in keeping with previous research, showing that fusifrom gyrus activation is related to processing of direct relative to averted gaze (George et al., 2001), and that amygdala activation has been shown to predict gaze toward fearful eyes (Gamer and Büchel, 2009) and associated with analysis of gaze cues when a person is actively monitoring for emotional gaze events (Hooker et al., 2003). Based on existing evidence, a model may be proposed whereby oxytocin modulates facial emotion recognition in part by activating the amygdala and fusiform gyrus to direct gaze shift towards the eye region. This model would benefit from further investigation using standardised measures of eye gaze and amygdala activation. Of note however, one more recent study showed no effect of oxytocin of gaze fixation (Lischke et al., 2012b).

### 4.4 Differential effects of oxytocin relative to genotype

There is now a robust body of evidence demonstrating effects of variation in the oxytocin receptor gene (OXTR) on a range of psychiatric conditions and behavioural measures (Bakermans-Kranenburg and van IJzendoorn, 2014; Meyer-Lindenberg et al., 2011). Both studies in our review examining effect of genotype (Montag et al., 2013; Sauer et al., 2012) demonstrated that variations in a single nucleotide polymorphisms (SNP) of OXTR (rs3796863 and rs401015 respectively) had a significant influence on and neuronal measures of facial emotion/social processing. In Sauer et al., homozygotic risk allele carriers showed slower reaction times and higher activation of left fusiform gyrus during visual processing of social stimuli. Oxytocin appeared to modulate this effect by enhancing activation differences between allele groups. Although Montag et al. (2013) did not observe a main effect of gaze direction on amygdala activity, genetic variation of the OXTR gene (rs401015) modulated right amygdala activity for direct > averted gaze under influence of OT.

Since these studies were published, novel methods (Chiu and Miller, 2016) and large-scale multicentre trials (Ripke et al., 2014) have come increasingly to the fore of molecular genetic research. Future studies examining the impact of genotype on the role of oxytocin will benefit from such approaches, which are more likely to contribute towards causal associations between genotype and neuroimaging and behavioural measures. Studies examining effects of individuals SNPs have yielded the most promising results when focusing on biological measures such as neuroimaging parameters (Bakermans-Kranenburg and van IJzendoorn, 2014). These are more likely to be successful than those focusing on more distal, complex social phenotypes with error-prone assessments (Bakermans-Kranenburg and van IJzendoorn, 2014).

### 4.5 Other considerations

It should be noted that many of the studies included in this review had small sample size (average 36.5 per group of healthy subjects) and while power calculations were inconsistently reported, many studies were likely significantly underpowered. The issue of low statistical power is a well-recognised criticism in both neuroimaging research (Button et al., 2013; Ioannidis, 2005) and studies involving oxytocin generally (Walum et al., 2016). Studies with low statistical power increase the likelihood of both type I and type II errors, producing poor quality research with ethical implications (Button et al., 2013). Factors which may address the issue of low statistical power include a priori power calculations, transparent disclosure of methods and findings where null findings are demonstrated, pre-registration of study protocols and analysis plans, and making study materials and data available, and working collaboratively to increase power and replicate findings (Button et al., 2013). Further, recent attempts for clearer guidance on power calculation (Durnez et al., 2016) and task design (Durnez et al., 2017) in fMRI studies should help improve quality of research in this area. It is notable that the GRADE score allocated by this review of 1 translates to ‘very low quality’ research, with particular problems due to sparse data, methodological concerns, and lack of agreement between studies.

A further consideration is the potential for publication bias in the published literature. Traditionally, meta-analysis can overcome the issue of statistical power, and can be used to assess publication bias. However, many of the studies returned from this review performed ROI analyses, which cannot be combined with whole-brain analyses in methodologically sound neuroimaging meta-analysis (Radua et al., 2012). Furthermore, meta-analysis requires that the contrasts in individual studies are testing similar constructs – in the current review, there were a relatively low number of studies (five) which could be grouped together. As such, a meta-analysis was not considered a worthwhile addition in this instance.

In conclusion, oxytocin modulates facial emotion processing in key ‘social brain’ areas in normal adults. This effect is mediated by individual differences, including sex and genotype. The evidence for differential response to facial emotions (with or without oxytocin) relative to implicit vs explicit tasks remains equivocal. Oxytocin's effects may be exerted in part through enhanced gaze processing, involving the amygdala and fusiform gyrus. Research in this area to date has been somewhat limited by low statistical power, methodological heterogeneity, and a dearth of multimodal studies. Future studies should ensure controls for individual differences, including sex, physiology, and genotype. Further work will benefit from accurate and transparent power calculation, collaborative projects involving genotyping and other methodologies.

## Funding

Dr John Tully is supported by a Wellcome Trust Clinical Research Training Fellowship. Grant no. 200099/Z/15/Z.
